# Thyroid dysfunction and risk of cutaneous malignant melanoma: a bidirectional two-sample Mendelian randomization study

**DOI:** 10.3389/fendo.2023.1239883

**Published:** 2023-11-29

**Authors:** Hua Dong, Lei Pan, Yanhui Shen, Qinxuan Xu, Jinyu Hu, Zhiwei Hu, Yuchang Fei

**Affiliations:** ^1^Endocrinology Department, Jiashan Hospital Affiliated of Jiaxing University, The First People’s Hospital of Jiashan, Jiaxing, Zhejiang, China; ^2^Department of Oncology, The First Affiliated Hospital of Zhejiang Chinese Medical University, Hangzhou, Zhejiang, China; ^3^Department of Traditional Chinese Medicine, Institute for Food, Drug and Product Quality Control of Jiaxing, Jiaxing, Zhejiang, China; ^4^Department of Integrated Chinese and Western Medicine, Jiashan Hospital affiliated of Jiaxing University, The First People’s Hospital of Jiashan, Jiaxing, Zhejiang, China

**Keywords:** thyroid dysfunction, hypothyroidism, hyperthyroidism, cutaneous malignant melanoma (CMM), Mendelian randomization study

## Abstract

**Background:**

Epidemiologic and observational data have found a risk association between thyroid dysfunction and cutaneous malignant melanoma (CMM), however, the cause and direction of these effects are yet unknown. By using a bidirectional two-sample Mendelian randomization (MR) methodology, we hoped to further investigate the causal link between thyroid dysfunction and CMM in this work.

**Methods:**

A genome-wide association study (GWAS) of 9,851,867 single nucleotide polymorphisms (SNPs) in a European population was used to develop genetic tools for thyroid dysfunction. Hypothyroidism was linked to 22,687 cases and 440,246 controls. For hyperthyroidism, there were 3545 cases and 459,388 controls. A total of 3751 cases and 372016 controls were included in the genetic data for CMM from UK Biobank (http://www.nealelab.is/uk-biobank) (the Dataset: ieu - b - 4969). Among them, inverse variance weighting (IVW) is the main MR Analysis method for causality assessment. MR-Egger method, MR Pleiotropic residual and outlier test (MR-PRESSO), and simple and weighted median (VM) were used to supplement the IVW method. Sensitivity analyses, mainly Cochran’s Q test, leave-one-out analysis, and MR Egger intercept test were performed to assess the robustness of the outcomes.

**Results:**

The two-sample MR Analysis results revealed a negative correlation between genetically predicted hypothyroidism and the probability of CMM (OR=0.987, 95%CI =0.075-0.999, *p*=0.041). The supplemental MR Analysis did not reveal any statistically significant differences, although the direction of the effect sizes for the other approaches was consistent with the IVW effect sizes. The results of the causal analysis were relatively robust, according to a sensitivity analysis. The risk of CMM was unaffected by hyperthyroidism (*p*>0.05). No correlation between CMM and thyroid dysfunction was seen in the reverse MR analysis.

**Conclusion:**

Although the magnitude of the causal association is weak and further investigation of the mechanism of this putative causal relationship is required, our findings imply that hypothyroidism may be a protective factor for CMM.

## Introduction

Hypothyroidism and hyperthyroidism are common endocrine disorders with potentially damaging health consequences that affect all populations worldwide. Among them, hypothyroidism affects 0.6%-12% of women and 1.3%-4% of men (1, 2); In the general population, the frequency of hyperthyroidism ranges from 0.2% to 1.3% (3–5), is 1.48 times more prevalent in women than in males (6), and rises with age. Thyroid hormones are essential for growth, neuronal development, reproduction, and regulation of energy metabolism (7). Increased blood thyroid-stimulating hormone (TSH) levels in an iodine-rich environment cause hypothyroidism, which is most typically caused by Hashimoto’s thyroiditis. Hypothyroidism is widely established to be related to comorbidities such as diabetes, and cardiovascular and cerebrovascular illness (8). Graves’ disease accounts for 70%-80% of hyperthyroidism in countries with adequate iodine and around 50% of hyperthyroidism in places with insufficient iodine (5, 9).

Since thyroid hormone is crucial for physiological processes like growth, maturation, and human metabolism, it has been suggested previously that thyroid function may influence the development of cancer (10, 11). With inconsistent findings from past research, the precise relationship between thyroid function and cancer has been a matter of controversy for more than 200 years (12, 13). CMM, the most dangerous type of skin cancer, is becoming more common everywhere. According to recent statistics, men and women in the United States are more likely to develop CMM than any other type of cancer (14). For a long time, scholars have recognized the “thyroid-skin connection”, but there are few studies on the role of THs in the occurrence and/or progression of CMM. Early observations found that both abnormally low and excessive thyroid hormones (THs) can change the appearance and function of human skin and its appendages, leading to pretibial myxoedema and alopecia at rest (15, 16). Thyroid-stimulating hormone receptor mRNA was found to be highly expressed in cultured keratinocytes, epidermal melanocytes, and melanoma cells by Andrzej (16). Genes associated with the hypothalamic-pituitary-thyroid axis are also expressed in the skin but are selective in both cell type and gene type. Giacomo et al. described a case of melanoma onset after utilizing hormone, thyroid and growth hormone replacement therapy (17). Shah Monica et al. showed in a retrospective study that male melanoma patients had a substantially higher frequency of hypothyroidism than the overall population (18). Although the results of current observational studies are useful for researching thyroid function and CMM they are prone to be influenced by several confounding variables, making it difficult to draw reliable conclusions about the cause of an event. Therefore, more research into the causes of thyroid illness and malignant cutaneous melanoma is required.

Mendelian randomization (MR) is a data analysis method applied to the etiology inference of epidemics, which uses genetic variation to assess the causal relationship between exposure and outcome. It serves as a valuable tool, especially when randomized controlled trials are not feasible to examine causal relationships and observational studies deviate due to confounding factors or reverse causal relationships, which can be addressed by using genetic variation as a tool variable for testing exposure. Since alleles follow the principle of random allocation during gametogenesis, genotypes can be used as instrumental variables of intermediate phenotypes to be studied to infer their causal relationship with disease states, and the estimated effect value is not affected by confounding factors and reverse causality (19, 20).

Genome-wide association studies (GWAS) that have been widely disseminated in recent years have made it possible for researchers to examine complicated disorders, and the findings of many features have paved the path for rigorous and well-researched MR Analysis. Numerous MR studies have examined the link between cancer and thyroid function level in recent years. According to Lu et al.’s research, hypothyroidism has a protective causative association that is inversely correlated with the likelihood of developing hepatocellular carcinoma (HCC) (21). TSH levels were found to be inversely correlated with thyroid cancer by Yuan et al.’s MR Analysis, and thyroid dysfunction was found to be related to breast cancer (22).

However, more MR analyses are required to further examine more reliable results as the GWAS database grows. This investigation used a two-sample MR analysis to determine the possible causative link between thyroid illness and CMM.

## Materials and methods

### Study design

**Figure 1:**
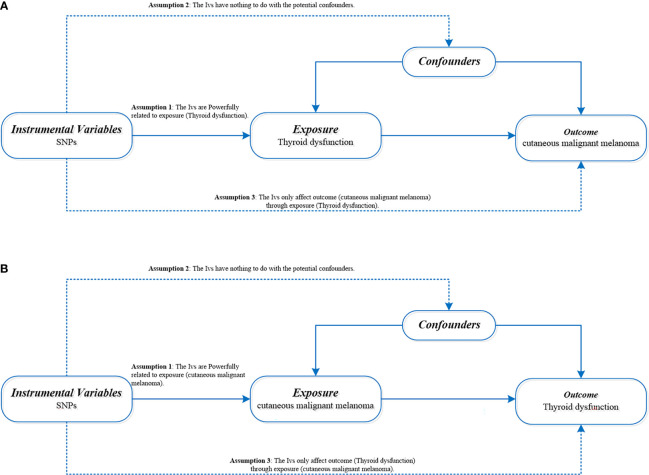
**(A)** Directed acyclic diagram of MR Framework to explore the causal relationship between thyroid dysfunction and CMM. **(B)** Directed acyclic graph of MR Framework to explore the causal relationship between CMM and thyroid dysfunction. SNPs, single nucleotide polymorphism; CMM, cutaneous malignant melanoma; iv, Instrumental variables.

Hypothyroidism and hyperthyroidism summary data are from genome-wide association studies (GWASs) from ieu open gwas project database (https://gwas.mrcieu.ac.uk/) retrieved. The data of hypothyroidism and hyperthyroidism were obtained from Dataset: ukb-b-20289 and Dataset: ukb-b-19732 with 9,851,867 single nucleotide polymorphisms (SNPS) in 462,933 Europeans. Hypothyroidism was associated with 22,687 cases and 440,246 controls. There were 3545 cases and 459,388 controls for hyperthyroidism. CMM of the data from the UK Biobank (http://www.nealelab.is/uk-biobank) (the Dataset: ieu-b-4969) with 3751 cases and 372,016 controls. A total of 11396019 SNPs were covered. All cases met the diagnostic criteria of hyperthyroidism/hypothyroidism and CMM (The diagnostic criteria are available in **Supplementary File 1**). **Table 1** shows the sources of data for the analysis. The exposure and outcome samples covered in this study were all human and were secondary analyses of previously published data. Therefore, ethical approval was not required. **Figure 1** shows three key assumptions of this bidirectional MR study.

**Table 1:** Sources of data for the analysis.

Phenotype		Source of Genetic Variants	
	Consortium	Participants	
Hyperthyroidism	MRC-IEU	Case	3545
		Control	459388
		Sample size	462933
		Number of SNPs	9851867
Hypothyroidism	MRC-IEU	Case	22687
		Control	440246
		Sample size	462933
		Number of SNPs	9851867
Cutaneous Malignant Melanoma	UK Biobank	Case	3751
		Control	372016
		Sample size	375767
		Number of SNPs	11396019

### Data sources and SNPs selection

We selected genome wide significant (*p*< 5 × 10 ^− 8^) single nucleotide polymorphism loci (SNPs) associated with CMM from large GWAS meta-analyses as instrumental variables (IV). Using thyroid dysfunction as the exposure factor and CMM as the outcome index, we found that there were 13 SNPs in the MR analysis of hyperthyroidism and CMM and 107 SNPs in the MR analysis of hypothyroidism and CMM. Using CMM as the exposure factor and thyroid disease as the outcome index, there were 8 SNPS in the MR Analysis of cutaneous melanoma and hyperthyroidism, and 10 SNPS in the MR Analysis of hypothyroidism and cutaneous melanoma. To ensure SNP independence, we performed LD-pruned tests (r^2^<0.001 and kb=10000) using R software.

### Statistical analysis

In this two-sample MR analysis, the bidirectional causality between thyroid dysfunction and CMM was estimated using the inverse-variance weighted (IVW), MR-Egger, simple and weighted median (VM), and MR Multiple effects residual and outlier test (MR-PRESSO).

IVW analysis is the main method of our MR research, which is characterized by regression without considering the existence of intercept term and using outcome variance because it has the most convincing estimation when directional pleiotropy of IVs is missing (23). MR-Egger is an alternative robust method for Mendelian randomization using summary data (24). At least 50% of the weight comes from valid IVs, then the weighted median will provide consistent estimates (25). MR-Egger and VM as complements should both be considered as sensitivity analyses for Mendelian randomization studies with multiple genetic variants. The MR-PRESSO method was used to identify horizontal pleiotropy outliers in multi-instrument summary-level MR testing and to reassess causal effects after removing pleiotropy IV (26).

**Figure 2:**
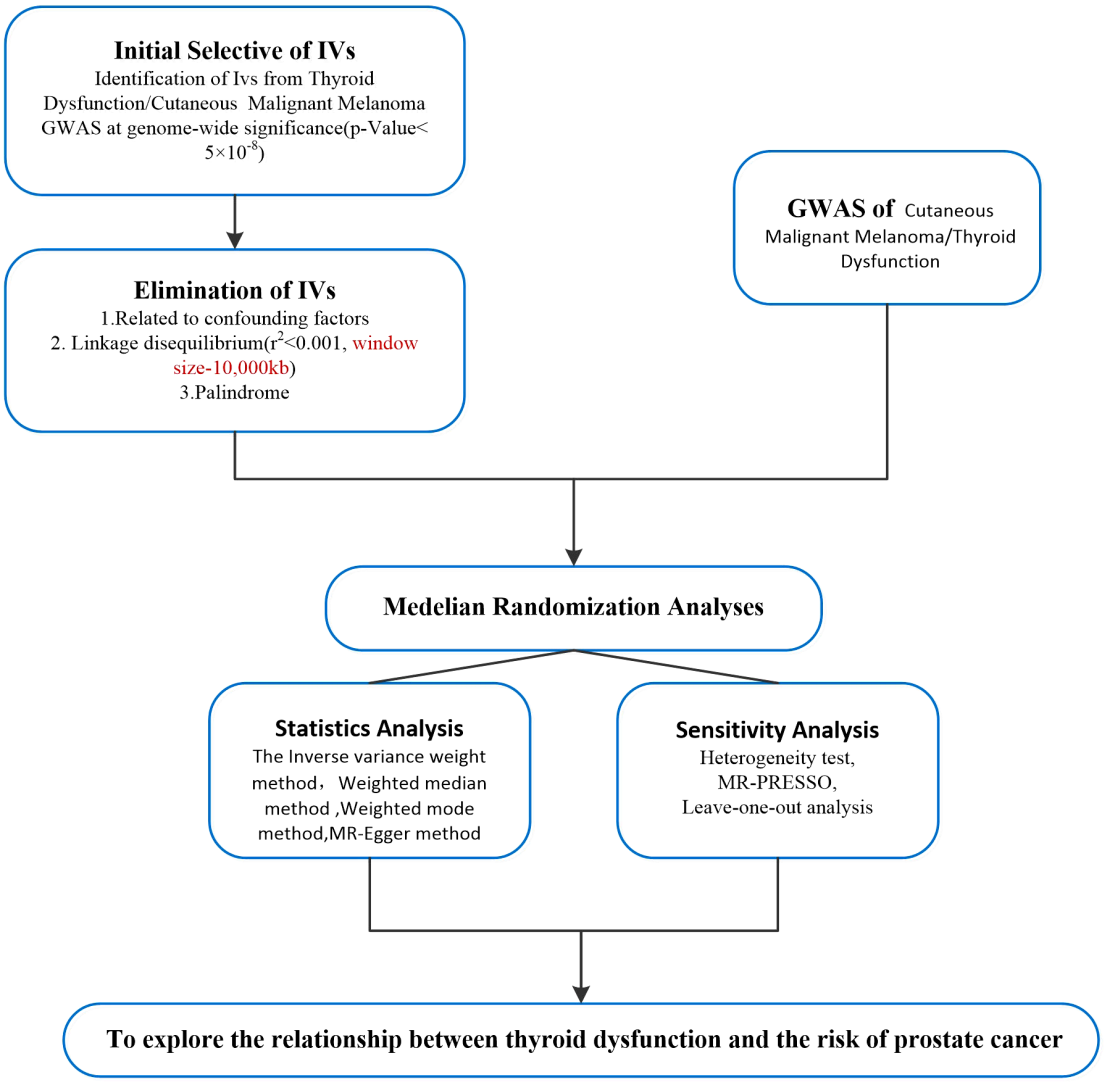
Flow chart of Mendelian randomization analysis of the study. iv, instrumental variable; GWAS, genome-wide association study; IVW, inverse variance weighted method; MR-PRESSO, Mendelian randomization pleiotropy residual sum, and outlier test; CMM, cutaneous malignant melanoma; iv, Instrumental variables.

To further account for possible horizontal pleiotropy, sensitivity analyses were performed to determine whether the results were robust or the data were heterogeneous. We used Cochran’s Q value to test heterogeneity (27), *p*<0.05 was considered to indicate the presence of heterogeneity, and IVW random effect method was used as the main effect size. The deviation of the intercept from zero in MR Egger regression is a valid indicator of response level pleiotropy. Leave-one-out analysis was performed by deleting an SNP in the analysis and estimating the causal effect (23). The flowchart for the selection of IVs and MR Analysis is shown in **Figure 2**.

We used the R packages “TwoSampleMR” and “MR-PRESSO” to do the MR analysis, and R software version 4.2.1 was used for all statistical analyses. R created a graphic depiction of the data.

## Results

### Positive MR relationship between thyroid dysfunction and CMM

A total of 107 SNPs were included as valid IVs for this analysis. IVW is MR’s method of meta-summarizing the effects of multiple loci when analyzing multiple SNPs. MR Analysis showed that hypothyroidism may increase the risk of CMM (IVW: OR = 0.987, 95%CI =0.075-0.999, *P*=0.041). IVW Cochran’s Q analysis of *p* < 0.05, indicates that there is heterogeneity between SNPs, therefore IVW random effect model was used as a main analysis method. MR Egger regression is used to detect the presence of horizontal pleiotropy in IVs. When the intercept test of MR Egger shows *p*<0.05, it indicates that the difference is statistically significant and there is horizontal pleiotropy. However, the results of MR Egger regression here show that *p*>0.05, the difference is not statistically significant, indicating that genetic level pleiotropy does not cause bias in the results (intercept=-0.000064, *p*=0.948).

**Figure 3:**
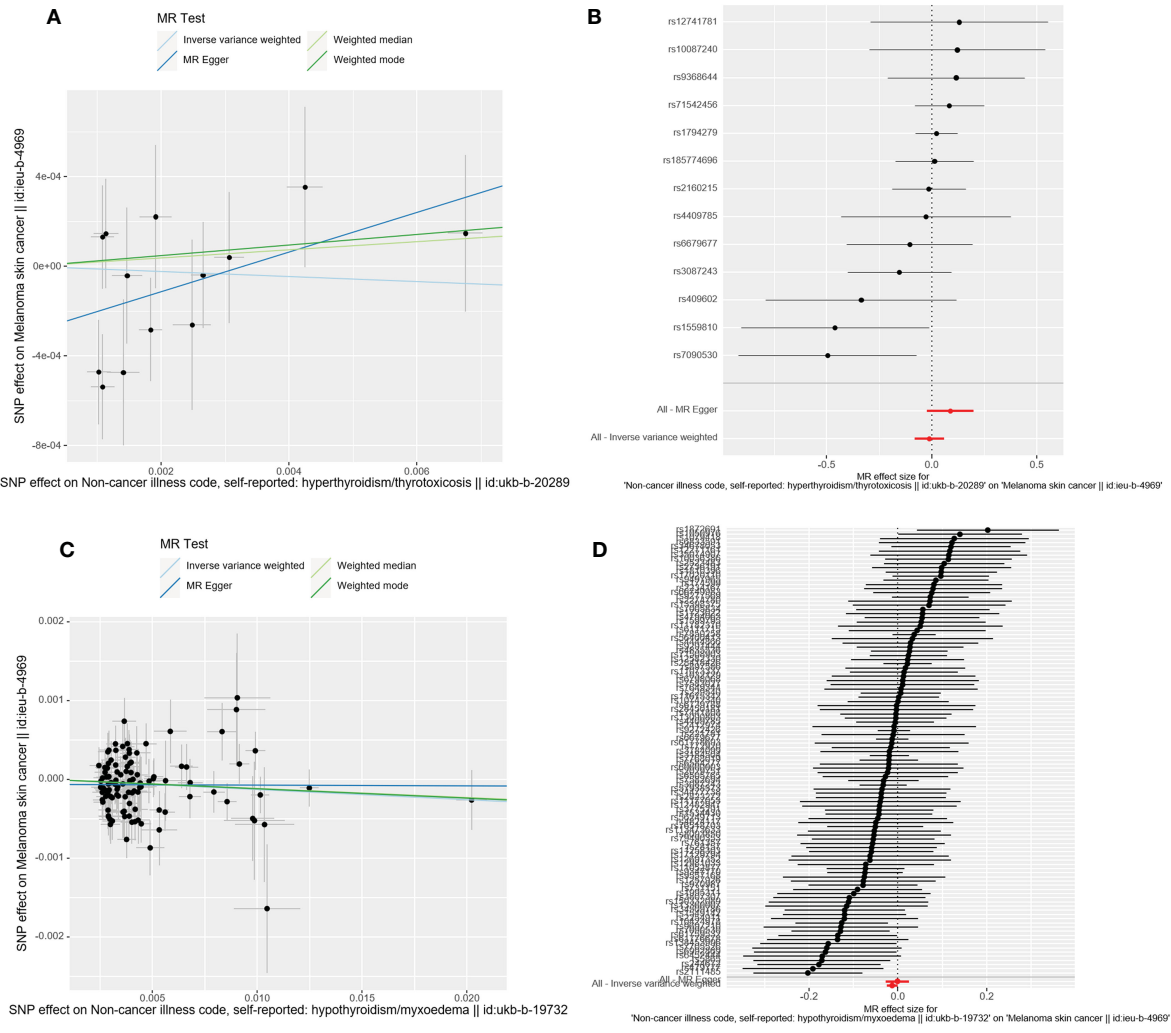
Scatter plot and forest plot of the causal relationship between thyroid dysfunction and CMM. CMM, cutaneous malignant melanoma. **(A)** Scatter plot of causality between hyperthyroidism and malignant cutaneous melanoma. **(B)** the susceptibility of hyperthyroidism to the risk of CMM; Red dots indicate the combined causal estimates for all SNPs using both MR-Egger and IVW) methods. **(C)** Scatter plot of causality between hypothyroidism and CMM. **(D)** the susceptibility of hypothyroidism to the risk of CMM; Red dots indicate the combined causal estimates for all SNPs using both MR-Egger and IVW) methods.

MR analysis showed that thyroid function hyperfunction and there is no causation between its CMM (IVW: OR = 0.989, 95% CI = 0.922 1.061, *p*=0.753). When hyperthyroidism was used as the exposure factor, Cochran’s Q analysis of IVW was *p*>0.05, so the IVW fixed effect model was used for MR Analysis. In addition, MR – the Egger intercept method (*p*=0.145) not detected the genetic level pleiotropic; No abnormal values were detected by the MR-PRESSO method (*p*=0.759).The specific results of the above MR Analysis and the results of the sensitivity analysis are shown in **Table 2** and **Figure 3**.

**Table 2:** MR analysis results.

MR estimates for the causal effect of thyroid dysfunction on Cutaneous Malignant Melanoma(CMM)										
	*Outcome*	*nSNP*	*IVW*		*MR-Egger*		*Weighted Median*		*Weighted Mode*	
			OR (95%CI)	*P*	OR (95%CI)	*P*	OR (95%CI)	*P*	OR (95%CI)	*P*
**Hyper**	CMM	13	0.989 (0.922,1.061)	0.753	1.093 (0.978,1.221)	0.145	1.018 (0.939,1.104)	0.658	1.024 (0.942,1.113)	0.588
**Hypo**	CMM	107	0.987 (0.975,1.000)	0.04	0.999 (0.973,1.026)	0.948	0.989 (0.971,1.007)	0.215	0.989 (0.969,1.007)	0.227
MR estimates for the causal effect of Cutaneous Malignant Melanoma(CMM) on thyroid dysfunction										
	*Outcome*	*nSNP*	*IVW*		*MR-Egger*		*Weighted Median*		*Weighted Mode*	
			OR (95%CI)	*P*	OR (95%CI)	*P*	OR (95%CI)	*P*	OR (95%CI)	*P*
**CMM**	Hyper	8	1.016 (0.955,1.080)	0.623	1.091 (0.945,1.259)	0.281	1.032 (0.954,1.116)	0.429	1.045 (0.956,1.143)	0.363
**CMM**	Hypo	10	0.811 (0.647,1.016)	0.068	0.838 (0.484,1.450)	0.545	0.832 (0.689,1.004)	0.055	0.808 (0.645,1.011)	0.096

### The relationship between CMM and thyroid dysfunction was examined by inverse MR

There was no evidence to support that CMM was a risk factor OR protective factor for hyperthyroidism and hypothyroidism (IVW: OR=1.016, 95%CI =0.955-1.080, *p*=0.623; OR=0.811, 95%CI= 0.647-1.016, *p*=0.068). Cochran’s Q test showed that there was heterogeneity among the 10 SNPs in the MR analysis of malignant cutaneous melanoma and hypothyroidism (*p*<0.05). Therefore, the IVW random effects model was chosen. Cochran’s Q test showed no significant heterogeneity of the 8 SNPs in the MR Analysis of CMM and hyperthyroidism (*p*=0.607), so we selected the IVW fixed effect model. We ultimately found no evidence to support a causal relationship between CMM and the risk of thyroid dysfunction.

**Figure 4:**
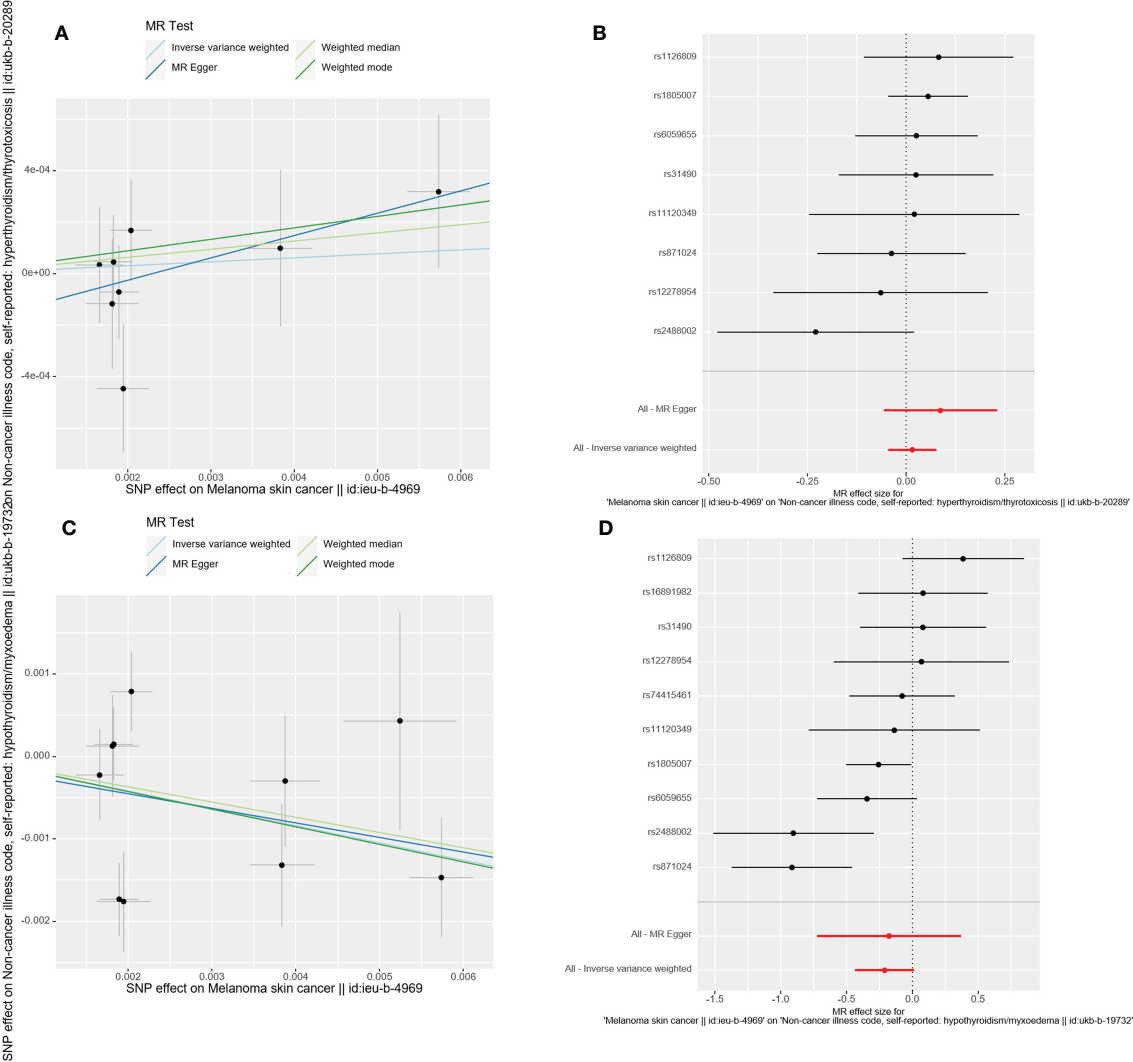
Scatter plot and forest plot of the causal relationship between CMM and thyroid dysfunction. CMM, cutaneous malignant melanoma. **(A)** Scatter plot of causality between malignant cutaneous melanoma and hyperthyroidism. **(B)** the susceptibility of CMM to the risk of hyperthyroidism; Red dots indicate the combined causal estimates for all SNPs using both MR-Egger and IVW) methods. **(C)** Scatter plot of causality between malignant cutaneous melanoma and hypothyroidism. **(D)** the susceptibility of CMM to the risk of hypothyroidism; Red dots indicate the combined causal estimates for all SNPs using both MR-Egger and IVW) methods.

Additionally, the outcomes of the IVW approach and the MR-Egger method, simple median method, weighted median method, and MR-PRESSO method were comparable (**Table 2** and **Figure 4**). The leave-one-out strategy added to the evidence of the reliability of the effect estimates from the MR Analysis (**Supplementary Materials-Figure 1**).

## Discussion

GWAS data from two distinct consortiums with populations of European ancestry were included in this two-sample MR Study. By using two-sample MR analysis, we methodically evaluated the bidirectional causality between thyroid insufficiency and CMM. Although there is a causal link between hypothyroidism and a lower risk of CMM, there is no causal link between hyperthyroidism and an increased risk of the disease. Similarly to this, there is no conclusive proof that there is a hereditary link between the risk of cutaneous melanoma and the occurrence of thyroid dysfunction, according to additional sensitivity analysis, and the effect estimates were reliable.

An autoimmune condition known as thyroid dysfunction includes both hyperthyroidism and hypothyroidism. Although the pathophysiology of the two diseases is distinct, clinical work makes it simpler to diagnose and treat the disease by enhancing the serological assessment of thyroid function in conjunction with clinical symptoms. However, growing clinical evidence in recent years has demonstrated that even small changes in thyroid function, such as subclinical dysfunction and changes within the reference range, can have a significant effect on clinical endpoint outcomes like bone mineral density, depression, metabolic syndrome, and cardiovascular disease (28). Cancer is one of the common diseases with the highest incidence rate and mortality in the world. The International Agency for Research on Cancer (IARC) predicts that there will be 18.1 million new cancer cases worldwide in 2020 (29). In addition, the American Cancer Society predicts that 1.95 million new cancer cases and 600,000 cancer deaths are expected in the United States in 2023 (30). Current research on the connection between thyroid health and cancer is contradictory. According to studies, there is no connection between SCH and breast or prostate cancer (12). The relative risk (OR) for cancer in 2,414,165 adults with diagnosed hypothyroidism compared to those without hypothyroidism was 1.73(1.72-1.74) based on the 2019 Spanish population-based statistics (*p*<0.0001). In addition, patients aged 65 years or older with hypothyroidism have a reduced risk of bladder, colorectal, gastric, pancreatic, and prostate cancers (31). Although existing studies have analyzed the causal relationship between thyroid dysfunction and other cancers, there has been no MR analysis of the causal relationship between thyroid dysfunction and CMM, and the exact relationship between the two has not been clarified by existing clinical and basic research.

Studies using epidemiological data have demonstrated a favorable correlation between hypothyroidism and the advancement of CMM (31–33). In line with the findings of the aforementioned epidemiological data, a retrospective analysis from the Israeli National Cancer Registry indicated that elevated Log-TSH was linked to a higher risk of CMM (HR: 1.11) (34). In contrast to the epidemiological data of the Israeli population mentioned above, our MR analysis, which was focused on a European population, revealed the opposite conclusion from a genetic point of view, which may have skewed the statistical results due to the Israeli population’s small size. In contrast to these investigations, our MR analysis comprised a considerably bigger population. Our work is more trustworthy because of our more stringent genetic instrument inclusion criteria and the removal of potential confounding variables. Furthermore, the aforementioned Israeli research discovered a link between hyperthyroidism and melanoma mortality (adjusted HR: 2.20) (35). Our MR analysis, however, could not identify a genetic link between hyperthyroidism and CMM. Since drug therapy alters the body’s initial immune status, more studies have recently focused on thyroid dysfunction in people with CMM caused by the use of immune checkpoint inhibitors (36), which is distinct from the causal relationship between thyroid dysfunction and CMM investigated in this study.

The advantage of our study is that we are the first to use a two-sample MR analysis to explore the bidirectional causal link between thyroid dysfunction and CMM. MR analysis is able to minimize the interference of confounding factors. Additionally, the genetic techniques that we developed by combining numerous separate data sets allowed us to reduce the possibility of biased outcomes as a result of under-enrollment. This MR analysis still has several limitations, such as the fact that the research population was chosen because it was European, which may have led to outcome bias. To generalize this analysis to other populations, more research is required.

We could have been unable to identify variations in thyroid dysfunction in the initial data set due to the heterogeneity of the included data sets.

## Conclusion

Our research demonstrates that hyperthyroidism does not raise the risk of CMM, whereas hypothyroidism is causally linked to a decreased risk of the disease. In addition, we found no clear evidence of genetic causality between CMM and the risk of thyroid dysfunction.

## Data availability statement

The **Supplementary Materials** section of this paper contains the original data and photographs used in this research. Please get in touch with the authors of this manuscript if you require any further original information.

## Ethics statement

The studies involving humans were approved by Medical Ethics Committee of the First People’s Hospital of Jiashan. The studies were conducted in accordance with the local legislation and institutional requirements. This study's exposure and result samples were human individuals, and it was a secondary analysis of already published data, thus no ethical approval was required. The Ethics Committee of the First People's Hospital of Jiashan granted us an exemption because all datasets utilized in this study were public domain.

## Author contributions

YF: Participating in the topic selection and writing of the study. HD: Writing the Manuscript. LP: Participating in the interpretation of the results of the MR Analysis. YS: Data Collection. JH and QX, ZH: Drawing the Figures and Tables. HD1†, LP2†, these authors contributed equally to this work and share first authorship. All authors contributed to the article and approved the submitted version.

## Supplementary material

Supplementary Figure 1 Leave-one-out plot of the risk of bidirectional causality between thyroid dysfunction and CMM. CMM, cutaneous malignant melanoma. **(A)** Hyperthyroidism and CMM; **(B)** Hypothyroidism and CMM; **(C)** CMM and hyperthyroidism; **(D)** CMM and hypothyroidism.

Supplementary Figure 2 Funnel plot of the risk of bidirectional causality between thyroid dysfunction and CMM. CMM, malignant cutaneous melanoma. **(A)** Hyperthyroidism and CMM; **(B)** Hypothyroidism and CMM; **(C)** CMM and hyperthyroidism; **(D)** CMM and hypothyroidism.
