# Supplementary material for: Thyroid dysfunction and risk of cutaneous malignant melanoma: a bidirectional two-sample Mendelian randomization study
Source: Front Endocrinol (Lausanne). 2023 Nov 29;14:1239883. doi: 10.3389/fendo.2023.1239883 (PMC10716543; doi:10.3389/fendo.2023.1239883)
Supplement: Supplementary file 1 [file DataSheet_1.docx]

**Supplementary File 1**

**Diagnostic criteria for hypothyroidism:**

At least one increase in thyrotropin and one decrease in free thyroxine were required.

**Diagnostic criteria for hyperthyroidism:**

The levels of T3, T4, FT3 and FT4 increased, while TSH decreased. Because most of hyperthyroidism is Graves' disease, which is an autoimmune disease of the thyroid, it is often accompanied by increased thyroid autoantibody, thyroglobulin antibody and thyroid peroxidase antibody, so the clinical test of thyroid stimulating hormone (TSH) receptor antibody-TRAb is positive.

**Diagnostic criteria for CMM：**

With few exceptions, the diagnosis of a benign or malignant melanocytic lesion does not depend on the staining characteristics of any particular immunohistochemical marker. Of the commonly used markers, Ki-67 is the best studied and probably the most useful marker for distinguishing nevus from melanoma. As a diagnostic tool, Ki-67 expression helps to distinguish nevi from malignant melanoma.
