# Supplementary figures and images for: Thyroid dysfunction and risk of cutaneous malignant melanoma: a bidirectional two-sample Mendelian randomization study
Source: Front Endocrinol (Lausanne). 2023 Nov 29;14:1239883. doi: 10.3389/fendo.2023.1239883 (PMC10716543; doi:10.3389/fendo.2023.1239883)

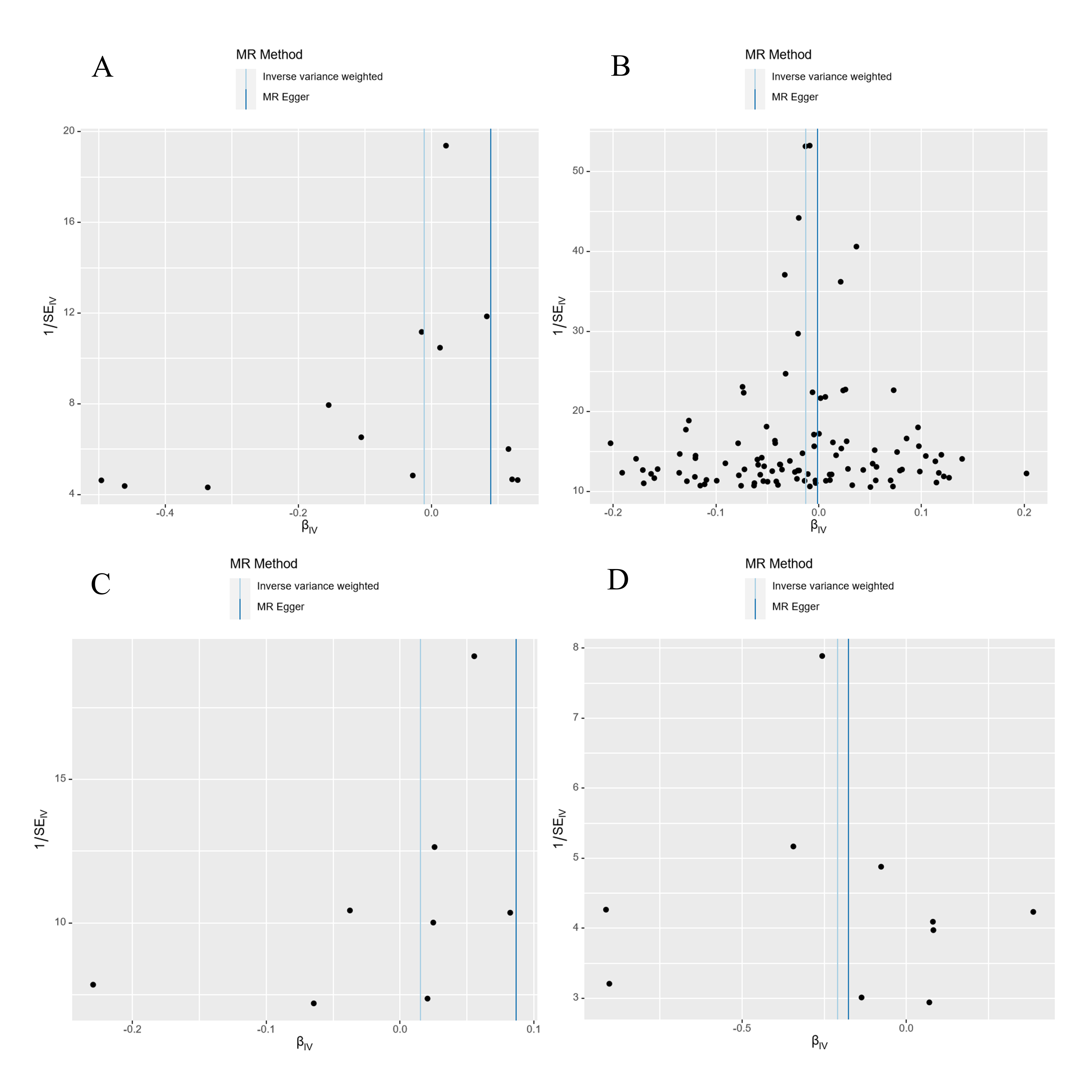

Supplement: Supplementary Figure 2 — Funnel plot of the risk of bidirectional causality between thyroid dysfunction and CMM. CMM, malignant cutaneous melanoma. (A) Hyperthyroidism and CMM; (B) Hypothyroidism and CMM; (C) CMM and hyperthyroidism; (D) CMM and hypothyroidism. [file Image_2.png]
